# Hémangioendothéliome épithélioïde de la cuisse: à propos d'un cas

**DOI:** 10.11604/pamj.2014.18.106.4403

**Published:** 2014-05-30

**Authors:** Ahmed Bardouni, Issam Elouakili, Younes Ouchrif, Redouane Ouakrim, Mohammed Kharmaz, Omar Lamrani, Farid Ismael, Abdo Lahlou, Mohammed Ouadaghirie, Mustapha Mahfoud, Mohammed Saleh Berrada, Mouradh El Yaacoubi

**Affiliations:** 1 Service de Traumatologie Orthopédie, CHU Ibn Sina, Rabat, Maroc

**Keywords:** hémangioendothéliome, épithélioïde, parties molles, tumeur vasculaire, hemangioendothelioma, Epithelioid, soft tissue, vascular tumor

## Abstract

L'hémangioendothéliome épithélioïde (HEE) est une tumeur vasculaire à malignité intermédiaire survenant essentiellement dans l'os, les tissus mous, le poumon et le foie. Souvent considéré comme une tumeur à malignité intermédiaire entre les hémangiomes bénins et les angiosarcomes agressifs, l'HEE comporte un potentiel de récidives locales, de métastases ganglionnaires et à distance et ne s'accompagne pas fréquemment de décès. Les auteurs rapportent l'observation d'un patient de 70 ans, Ayant présenté un hémangioendothéliomeépithélioïde des parties molles de la cuisse avec un recul de 1an sans récidive.

## Introduction

L'hémangioendothéliome épithélioïde (HEE) est une tumeur vasculaire à malignité intermédiaire survenant essentiellement dans l'os, les tissus mous, le poumon et le foie. Souvent considéré comme une tumeur à malignité intermédiaire entre les hémangiomes bénins et les angiosarcomes agressifs, l'HEE comporte un potentiel de récidives locales, de métastases ganglionnaires et à distance et ne s'accompagne pas fréquemment de décès [1–4]. A propos d'une observation d'HEE des tissus mous, nous proposons de rappeler les caractéristiques anatomo-cliniques de cette tumeur en insistant sur les aspects anatomo-pathologiques et le traitement.

## Patient et observation

Il s'agit d'un patient âgé de 70 ans, sans antécédents pathologiques particuliers, qui s'est présenté en consultation pour une masse volumineuse, non douloureuse de la cuisse gauche découverte depuis 2 ans et ayant augmenté progressivement de volume. Aucun autre signe fonctionnel n'a été rapporté par le patient notamment l'altération de l’état général.

**Figure 1:**
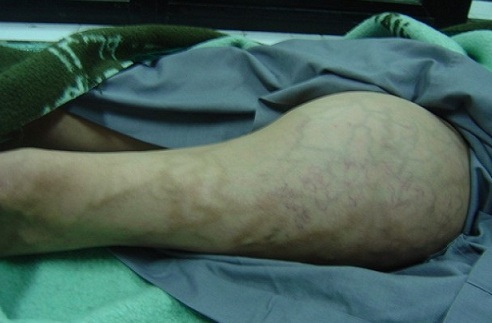
Masse de 25cm, bien limitée, avec présence de circulation collatérale en regard, sans signes inflammatoires associés

L'examen clinique objectivait une masse dont le grand axe était d'environ 25cm, bien limitée, non sensible, mobile par rapport au plan superficiel et adhérente au plan profond faisant corps avec le muscle quadriceps, avec présence de circulation collatérale en regard, sans signes inflammatoires associés (Figure 1). Les aires ganglionnaires étaient libres, notamment celles de la région inguinale. Le reste de l'examen clinique était sans particularités.

**Figure 2:**
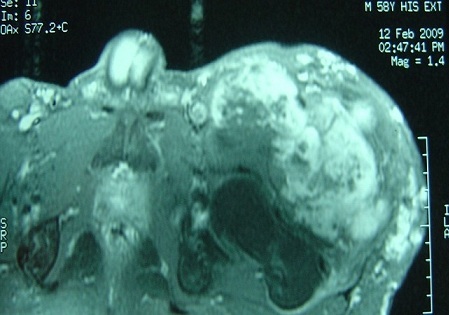
IRM du membre inférieur gauche en coupe transversale objectivant un processus expansif envahissant le muscle quadriceps

**Figure 3:**
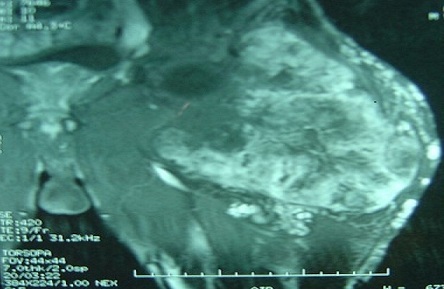
Coupe frontale du processus tumoral

Des radiographies standards du membre inférieur gauche ne montraient pas d'anomalies osseuses. Une IRM a été réalisée chez le patient révélant un processus expansif au dépend du muscle quadriceps, bien limité, de contours polylobés, avec des plages hétérogènes prenant le contraste après injection de Gadolinium (Figure 2, Figure 3).

**Figure 4:**
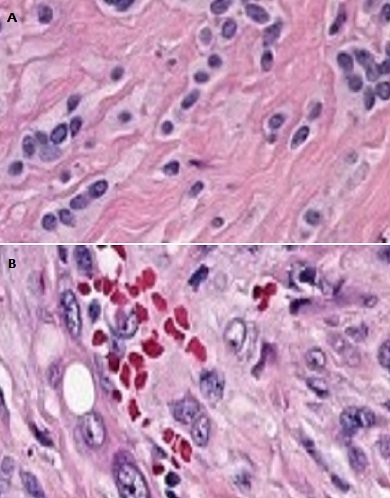
A) Hémangioendothéliome épithélioïde composé de nids et de cordons de cellules endothéliales épithélioides distribuées dans un stroma myxohyalin; B)Atypies cellulaires significatives

Le bilan d'extension à distance, comportant une radiographie thoracique et une TDM thoraco-abdominale, n'a pas montré d'adénopathies ni de localisations secondaires au niveau pulmonaire ou hépatique. L'indication chirurgicale a été retenue. Une biopsie initiale a été réalisée, suivie d'une exérèse large de la tumeur emportant tout le muscle vaste externe. L'examen anatomo-pathologique de la pièce opératoire a conclu à un hémangioendothéliome épithélioïde (Figure 4).

Les suites opératoires étaient simples. Le suivi du patient a été régulier avec un recul d'une année. Nous n'avons noté aucune récidive loco-régionale ni métastase à distance au cours de cette période. Le patient a conservé une fonction normale du membre opéré.

## Discussion

L'hémangioendothéliome épithélioïde (HEE) des tissus mous est une tumeur d'origine vasculaire rare et à malignité intermédiaire. Le terme d'HEE a été initialement proposé par Weiss et Enzinger en 1982 [5] pour désigner un groupe hétérogène de tumeurs vasculaires présentant des caractéristiques cliniques, histologiques et évolutives qui sont intermédiaires entre les hémangiomes et les angiosarcomes. Il s'agit donc de tumeurs «borderline» [1–3, 6].

L'HEE a été décrit au niveau de l'os, des tissus mous et d'autres organes: poumon, foie, rate, cerveau, sein, cœur [3, 6, 7].

**Epidemiologie**: Sur le plan épidémiologique, l'HEE des tissus mous est rare. Il est observé chez l'adulte d’âge moyen, rarement au cours de l'enfance et aussi bien chez l'homme que chez la femme. Dans une série de 49 cas d'hémangioendothéliome des parties molles colligées entre 1989 et 2005 [8], l’âge moyen des patients était de 49 ans avec des extrêmes entre 9 et 93 ans. 21 patients étaient des hommes et 28 étaient des femmes.

**Etude Clinique**: L'hémangioendothéliome se caractérise par une latence clinique avec un délai de consultation allant jusqu’à 10 ans voire plus chez le tiers des patients [2, 4, 5, 9, 10]. Typiquement, les patients ayant un hémangioendothéliome présentent des masses infiltrantes, uninodulaires ou multinodulaires, de siège dermique ou sous cutané, dont la croissance se fait de manière très lente. Ces masses siègent dans la majorité des cas au niveau des extrémités distales, comme dans notre cas. Elles ont tendance à se regrouper en grappe dans une seule région. La tête, la nuque, le thorax et l'abdomen ont été aussi rapportés comme étant des sites d'origine de la tumeur. Dans de rares circonstances, les lésions peuvent être multifocales [1, 4, 11–13]. Ces lésions causent rarement des modifications de la coloration de la peau en regard. Ceci prête à confusion avec certaines tumeurs non vasculaires. Cependant, une coloration rouge-brunâtre est fortement évocatrice d'un hémangioendothéliome à cellules fusiformes [10, 14, 15]. Une symptomatologie vasculaire peut s'associer à type d’œdème et de thrombophlébite [4]. Des adénopathies peuvent être retrouvées au niveau des territoires de drainage du site de la tumeur [16].

### Histologie

Dans au moins 50% des cas, la tumeur prend naissance à partir d'un petit vaisseau qui est souvent de nature veineuse. Exceptionnellement, elle se développe à partir d'une grosse veine ou artère [1, 4].

**Macroscopie**: La tumeur est soit blanc-rougeâtre soit blanc-grisâtre toujours mal limitée en périphérie [3, 12]. Les calcifications sont rarement observées surtout dans les localisations profondes [1, 2, 4].

**Microscopie**: Selon la nouvelle version de la classification des tumeurs des tissus mous de l'Organisation Mondiale de la Santé (OMS), l'HEE se définit histologiquement comme une tumeur vasculaire angiocentrique, constituée de cellules endothéliales épithélioïdes dans un stroma fibro-myxoïde [4] (Figure 4). L'aspect angiocentrique est caractéristique et présent dans 50% des cas.

Les cellules tumorales s’étendent de façon centrifuge de la lumière d'un gros vaisseau vers le tissu de voisinage [1, 2, 4, 9]. Cette lumière contient des cellules tumorales, des débris nécrotiques et du collagène dense. Les cellules tumorales se disposent de façon isolée ou se groupent en cordons courts ou en petits amas. Elles sont arrondies, ovalaires désignées comme « épithélioïdes » ou « histiocytoïdes » ou légèrement fusiformes [4, 5, 9]. La différentiation vasculaire primitive est observée à l’échelle cellulaire. Elle se traduit par la présence de petites lumières vasculaires intra-cytoplasmiques qui peuvent contenir parfois des globules rouges. [2, 4, 5, 9](Figure 5). Ces cellules sont entourées par un stroma myxoïde ou parfois hyalin. Les atypies cytonucléaires sont légères voire absentes et l'activité mitotique est faible ou nulle [4]. Des foyers d'ossification métaplasique ainsi que des cellules géantes multinucléées sont souvent observés dans les localisations profondes [4]. Certains signes histologiques sont corrélés avec une évolution tumorale plus agressive: la présence d'atypies, de foyers de nécrose, de mitoses > 1mitose par 10 champs au fort grossissement (X40) et de foyers de cellules fusiformes [2, 4].

Certains auteurs ont proposé le terme d'HEE malin ou atypique en présence de ces signes histologiques péjoratifs [4].

Ces derniers sont retrouvés dans 30% des cas des HEE. Une autre étude ayant porté sur 49 cas a montré que la taille tumorale et l'activité mitotique sont associées à un risque de mortalité élevé. Par contre, le site tumoral, les atypies cytologiques, la présence de nécrose et l'aspect fusiforme des cellules n’étaient pas des facteurs de mauvais pronostic [8].

**Etude Genetique**: Une étude cytogénétique de 2 cas d'HEE, localisés dans le foie et les tissus mous, a permis de mettre en évidence la présence d'une translocation entre les chromosomes 1 et 3 (t(1;3)(p36.3;q25) qui semble être potentiellement caractéristique de l'HEE [1, 6].

**Traitement**: Le traitement de l'hémangioendothéliome doit être le plus conservateur possible en raison de son comportement clinique bénin. Plusieurs options thérapeutiques ont été proposées notamment la chirurgie, la corticothérapie systémique, la cryothérapie, la destruction au laser, la radiothérapie, la chimiothérapie ainsi que l'embolisation sélective. L'interleukine 2 recombinante, un dérivé des lymphokines des lymphocytes T (activation des cellules T cytotoxiques et des cellules Natural killers) a été essayée avec succès [14]. Le traitement chirurgical est particulièrement difficile à cause de la mauvaise délimitation périphérique de la lésion et de l'infiltration diffuse des muscles, des tendons et des structures vasculo-nerveuses sous jacentes. Ainsi, la résection doit être la plus large possible [12]. C'est ce qui a été réalisé chez notre patient. Le traitement de l'HEE de bas grade de malignité repose essentiellement sur la résection marginale avec une surveillance stricte compte tenu de la possibilité des récidives locales et du risque des métastases à distance. La radiothérapie adjuvante est surtout indiquée dans les formes multicentriques et semble être efficace. Le traitement des formes de haut grade de malignité consiste en une chirurgie plus radicale. La polychimiothérapie utilisée dans les formes agressives multifocales, n'a pas d'efficacité clairement démontrée [11]. Néanmoins, une étude a rapporté le succès d'une cure par Cisplatine à faible dose et de radiothérapie modérée sans recours à la chirurgie [17]. Cependant, la radiothérapie doit être découragée en raison de son incrimination dans la transformation sarcomateuse de la tumeur avec risque accru de métastases [1, 14].

**Evolution**: L’évolution étudiée dans une série de 46 cas d'HEE des tissus mous [18], a révélé la survenue de 6 cas de récidives tumorales (13%), 14 cas de métastases (31%) et 6 décès (13%) [2]. Moins de 50% de ces malades présentant des métastases vont décéder de leur maladie. La plupart des métastases sont localisées aux ganglions locorégionaux. Les récidives et les métastases à distance sont surtout l'apanage des HEE atypiques [1, 2]. En effet, les formes classiques et malignes d'HEE présentent un taux de récidive locale de 10-15%, de métastases: 20-30% et de mortalité: 10-20%. Ceci implique que les signes histologiques de mauvais pronostic sont associés à un risque accru de métastases. A l'opposé, certaines tumeurs sont assez régulières histologiquement mais peuvent donner lieu à des métastases. De ce fait, le pronostic est difficile à prédire sur les aspects histologiques et on peut considérer que l'HEE correspond à un spectre de lésions vasculaires épithélioïdes qui représentent différents stades de différenciation au lieu d'entités tumorales distinctes bien définies [1, 9]. La survie des patients présentant un hémangioendothéliomeépithélioïde des parties molles a été analysée en fonction des facteurs de risque, notamment, la taille tumorale > 3cm et les mitoses > à 3/50 champs [8]. Il s'est avéré que la survie globale à 5 ans des patients était de 81%. Elle est de 100% lorsqu'il n'y a pas de facteurs de risque. En présence de ces derniers, la survie à 5 ans est seulement de 59%. Dans notre cas, le suivi du patient a été régulier avec un recul d'une année. Nous n'avons noté aucune récidive loco-régionale ni métastase à distance au cours de cette période. Le patient a conservé une fonction normale du membre opéré.

## Conclusion

L'hémangioendothéliomeépithélioïde (HEE) des tissus mous est une tumeur rare d'origine vasculaire considérée comme étant une tumeur à malignité intermédiaire. Elle comporte donc, un risque de récidive locale, d'envahissement ganglionnaire et de métastase à distance. Cliniquement, la tumeur reste longtemps asymptomatique.

Son expression clinique est généralement fruste. Sur le plan histologique, il s'agit d'une tumeur vasculaire angiocentrique, constituée de cellules endothéliales épithélioïdes dans un stroma fibro-myxoïde. Sur le plan immunohistochimique, la tumeur exprime les marqueurs endothéliaux, notamment, le CD31, ce qui confirme l'origine vasculaire des cellules tumorales. Le traitement est essentiellement chirurgical et doit être le plus conservateur possible. La chimiothérapie systémique peut être de mise dans les formes agressives multifocales ou si la tumeur s'accompagne de métastases.
